# Experimental Study of Glow Discharge Polymer Film Ablation with Shaped Femtosecond Laser Pulse Trains

**DOI:** 10.3390/ma18204761

**Published:** 2025-10-17

**Authors:** Qinxin Wang, Weiwei Xu, Xue Wang, Dandan Shi, Jingyuan Wang, Liyan Zhao, Yasong Cui, Mingyu Zhang, Jia Liu, Zhan Hu

**Affiliations:** 1 School of Electrical and Information Engineering, Jilin Engineering Normal University, Changchun 130052, China; xuww@jlenu.edu.cn (W.X.); xwzavw@163.com (X.W.); 1131854@jlenu.edu.cn (D.S.); wjingyuancc@163.com (J.W.); zly981207@163.com (L.Z.); zidiao1983@126.com (Y.C.); mingyuzmy@jlenu.edu.cn (M.Z.); liujia@jlenu.edu.cn (J.L.); 2 Institute of Atomic and Molecular Physics, Jilin University, Changchun 130012, China

**Keywords:** femtosecond laser processing, pulse shaping, microhole fabrication, laser–material interaction

## Abstract

A glow discharge polymer (GDP) has unique physical properties—transparency, brittleness, and hardness—that pose challenges for traditional mechanical machining techniques. We have investigated the microhole fabrication of GDP films using shaped femtosecond laser pulses to study the influence of pulse shape, delay between subpulses, and focusing position on processing precision and efficiency. By precisely controlling pulse characteristics, such as duration, energy, and subpulse intervals, the efficiency, hole morphology, and processing quality were significantly improved. The experimental results demonstrated that femtosecond lasers with subpulses produce smaller and more uniform microholes compared to transform-limited pulses. Furthermore, both the pulse shape and focusing position of the laser were found to further influence ablation efficiency. This study establishes, for the first time, the critical role of temporal pulse shaping in optimizing the femtosecond laser drilling of GDP films, which provides valuable information on optimizing femtosecond laser parameters for precision processing of polymer films and advances the potential for microhole fabrication in industrial applications.

## 1. Introduction

Ultrafast laser processing is a powerful tool in modern material processing, offering minimal thermal damage and precision ablation benefits from its ultra-short pulse durations [1,2,3,4,5]. The ability to manipulate femtosecond laser pulses has led to significant advancements in micro- and nanomachining, enabling the creation of intricate structures with high precision and minimal distortion [6,7,8,9]. Among the various methods of femtosecond laser processing, shaping the temporal behavior of laser pulses has become a key strategy to improve the efficiency and quality of ablation in materials, such as metals, semiconductors, polymers, and biological tissues [10,11,12,13,14,15,16]. By altering the pulse shape, including the pulse duration and the number of subpulses, it is possible to control the energy deposition during the interaction between the laser and material [13,17,18,19]. Furthermore, the ability to precisely control pulse duration and intensity significantly enhances the versatility of femtosecond lasers in a wide range of applications, including microhole drilling, surface structuring, and material removal [20,21]. Noël et al. reported that increasing the time delay between two pulses led to stronger fluorescence signals, shallower ablation holes, and fewer nanoparticles on the sample surface [22]. In another study, Qi et al. investigated femtosecond laser drilling of aluminum film under vacuum conditions using shaped pulse sequences. They found that, at higher pulse energies, a three-pulse train improved drilling efficiency, and longer subpulse intervals resulted in smaller hole diameters [17]. Additionally, tailored pulse shaping helps to mitigate unwanted thermal effects and structural damage, an essential advantage when processing materials with complex properties, such as transparent or soft materials, where precise ablation control is critical [6,23,24,25].

A glow discharge polymer (GDP) ball can be used as a container in laser-induced nuclear fusion, and injecting reactant fuel makes the research of drilling holes in GDP essential. GDP is a transparent, hard, and brittle material composed of carbon and hydrogen [26,27,28]. Usually, GDP films were selected as a model system for this fundamental study due to their simplified geometry, which facilitates the analysis of laser–plasma interaction mechanisms. The findings are expected to provide valuable insights for optimizing processes on more complex GDP-based targets, such as spherical shells used in laser fusion experiments. The GDP film has several advantageous properties, including high density without pinholes, excellent thermal stability, strong insulating characteristics, and infrared transparency. These features enable GDP films to effectively minimize initial perturbations during the implosion process, thus suppressing Rayleigh-Taylor instabilities under intense laser irradiation. Moreover, GDP films are especially valued in high-energy physics and precision optics for their outstanding surface smoothness and structural stability [29,30]. Conventional mechanical micro-machining methods have significant limitations when applied to such materials due to their inherent physical properties [27]. With advances in laser technology, femtosecond lasers have become increasingly prevalent for fabricating microholes in these materials. Modulated surface patterns can be produced by rastering a focused femtosecond laser across discrete points on the capsule surface, delivering a controlled number of pulses at each location [31]. By tuning laser parameters such as pulse width, energy, and repetition rate, researchers have achieved high-quality micro-machining products. Furthermore, recent work has shown that shaping the temporal profile of laser pulses can significantly enhance both machining efficiency and ablation quality [21,32,33].

This study investigates the ablation of GDP films using femtosecond laser pulses with various temporal shapes, including different pulse durations and subpulse configurations. The results reveal that ablation efficiency, hole morphology, and overall processing quality can be significantly improved by precisely controlling pulse characteristics, such as duration, energy, and subpulse intervals. Notably, a three-pulse train with a 0.3 ps subpulse interval, focused within the film, yields the highest ablation quality. The purpose of this study was to establish temporal pulse shaping as a novel method for precision machining of GDP films, enabling control over laser–plasma interaction to produce high-quality microholes.

## 2. Experimental Methods and Setup

**Figure 1:**
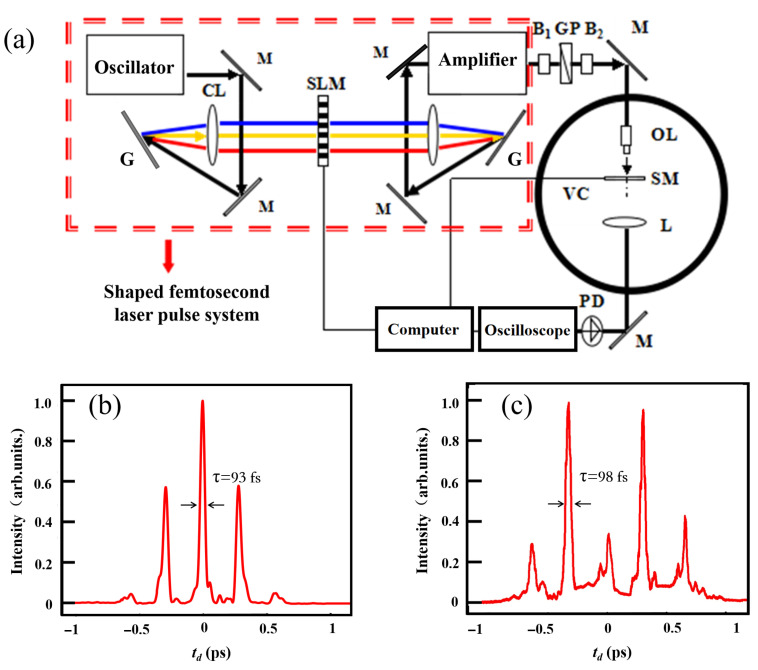
(**a**) Schematic diagram of the shaped femtosecond laser vacuum ablation system. (The meanings of the symbols are as follows: G: grating, CL: cylindrical lens, SLM: spatial light modulator, M: 800 nm high-reflection mirror, GP: Glan prism, $B_{1}$: half-wave plate, $B_{2}$: quarter-wave plate, VC: vacuum chamber, OL: focusing objective lens, SM: sample, L: optical lens, and PD: photodiode). (**b**,**c**): The measured pulse shapes of three-pulse and five-pulse sequences used for laser ablation.

The experiments were performed in a custom-built femtosecond shaping laser ablation system [14,18], as shown in Figure 1a. The femtosecond laser from a Ti: sapphire chirped pulse amplification system (Spitfire, Spectra physics) outputs the 90 fs pulses with a central wavelength of 800 nm under a repetition rate of 1 kHz, with a maximum energy of approximately 1 mJ per pulse. To precisely control the laser’s ablation on materials, a femtosecond pulse shaping was built. The 4f-shaper was placed between the laser oscillator and the amplification to avoid the damage of the liquid crystal spatial light modulator. The laser pulse from the oscillator (Tsunami, Spectra physics) was diffracted by a grating, which was then focused onto the Fourier plane of a spatial light modulator (SLM, CRI, model 640-D-VN). Then, the laser was compressed by passing through a second pair of lenses and grating. The shaped seed pulses were amplified to generate higher-energy shaped femtosecond pulses after passing through the CPA system. During the drilling process, linearly polarized laser light was converted to circular polarization to produce cleaner and more symmetrical holes [34]. For all measurements performed under different pulse shapes (e.g., single-pulse vs. multipulse), the total energy fluence was kept constant at 25.5 J/cm^2^ to study the effects of temporal pulse shaping. Experiments were conducted in a low-vacuum environment (1 Pa). The laser was focused onto the GDP film using a lens (Edmund NT59-877). The focused beam characteristics, including the beam diameter (1.96 µm) and Rayleigh length (24.2 µm), were quantified using the knife-edge method. The 120 µm GDP samples were mounted on a 3D translation stage and repositioned after each drilling event. The transmission signals of the laser passing through the film were collected using a lens and photodiode (Thorlabs DET10A). The resulting microhole morphology was analyzed using an optical microscope (Olympus BX51).

**Figure 2:**
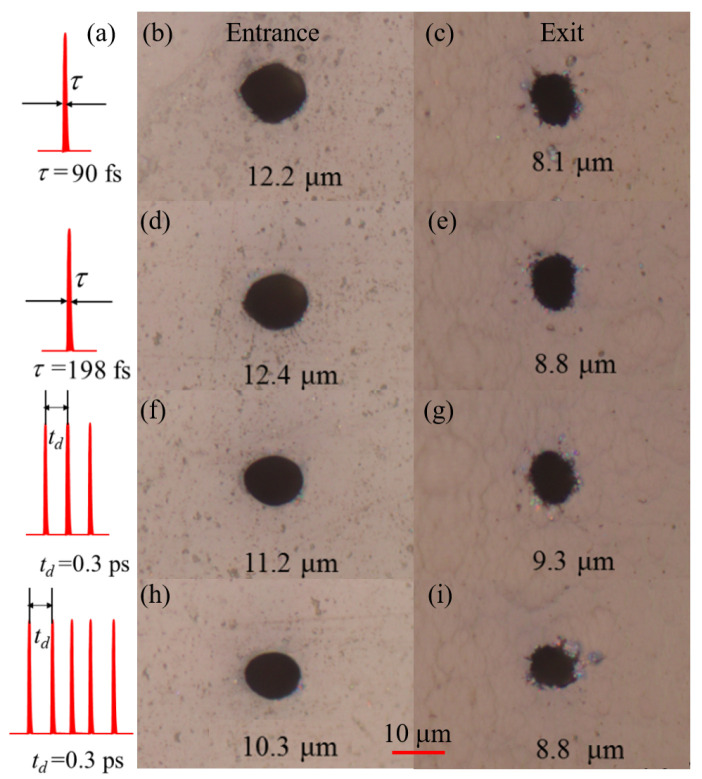
Different pulse shapes are used for drilling under intensity of 25.5 J/${\mathrm{cm}}^{2}$. The first column shows the pulse shapes (**a**), and the subsequent columns (**b**,**d**,**f**,**h**) show the entrance images obtained from the corresponding shaped pulse drilling; (**c**,**e**,**g**,**i**) present the corresponding exit morphology images. A total of 60,000 laser shots are used for each experiment.

The temporal profiles of the laser pulses—controlled via phase patterns applied to a spatial light modulator (SLM)—were controlled using a computer. The pulse shapes used in the experiments include single pulses with varying durations, triple pulses (TPs), and five pulses (FPs). The corresponding results are presented in Figure 2. For comparison, drilling with a transform-limited pulse was also performed. A broadened pulse with a duration of approximately 200 fs was generated by applying a second-order spectral phase. Multipulse shaping was achieved by imposing a sinusoidal phase function on the laser spectrum [35,36], described by

(1) $$\Phi =A\sin\left[\frac{2\pi }{T}\left(n-n_{0}\right)+\phi \right],$$

where A is the amplitude of the phase modulate, T is the period, n and $n_{0}$ are the pixel positions on SLM, and ϕ is the initial phase. The T value changes the delay between different peaks in the shaped pulse, and the delay can be calculated with $t_{d}=\frac{17.44}{T}$. The modulation amplitude A controls the number of subpulses: a TP shape is produced with A = 1.2566, while an FP shape is achieved with A set to 2.8274. All pulse shapes used in the ablation experiments were measured and characterized using multiphoton intrapulse interference phase scan technique [37,38]. The corresponding temporal intensity profiles of the TP and FP pulses are measured before the focus lens, and transmission through the focus lens only contributes to second-order dispersion, which will not change the pulse shape significantly. The results shown in Figure 1b,c, respectively, demonstrate excellent agreement with those predicted by the multipulse shaping method.

## 3. Results and Discussion

### 3.1. Influence of Pulse Shape on Laser Processing

Figure 2b–i show the entrance and exit images of microholes drilled using different-shaped femtosecond laser pulses. With a Fourier transform-limited pulse, the resulting hole had an entrance diameter of 12.2 µm and an exit diameter of 8.1 µm, yielding a diameter difference of 4.1 µm. When the pulse duration was extended to 200 fs, the diameters of the entrance and exit increased slightly to 12.4 µm and 8.8 µm, respectively. For the TP and FP configurations, the subpulse interval was set at 0.3 ps (corresponding to T = 60). Drilling with the TP laser produced a hole with an entrance diameter of 11.2 µm and an exit diameter of 9.3 µm, resulting in a much smaller diameter difference of 1.9 µm. When using the FP laser, the entrance and exit diameters were further decreased to 10.3 µm and 8.8 µm, with a difference of 1.5 µm. The enhanced drilling performance observed for the temporally shaped pulse (with a subpulse interval of 0.3 ps) is likely due to more efficient laser–energy coupling and confined plasma dynamics. The prepulse initiates plasma formation, and the subsequent subpulses effectively deposit their energy into this preformed plasma, leading to enhanced and spatially confined energy deposition. This process results in more precise ablation and the formation of holes with smaller diameters and reduced difference between the entrance and exit diameters. The FP laser, while producing the smallest holes, required a significantly higher number of pulses to achieve breakthrough (∼4400 shots) compared to the TP laser (∼2000 shots), reducing its processing efficiency. The evaluation of optimal performance therefore depends on the specific application requirements between precision and speed.

### 3.2. Effect of Focus Position on Laser Processing

**Figure 3:**
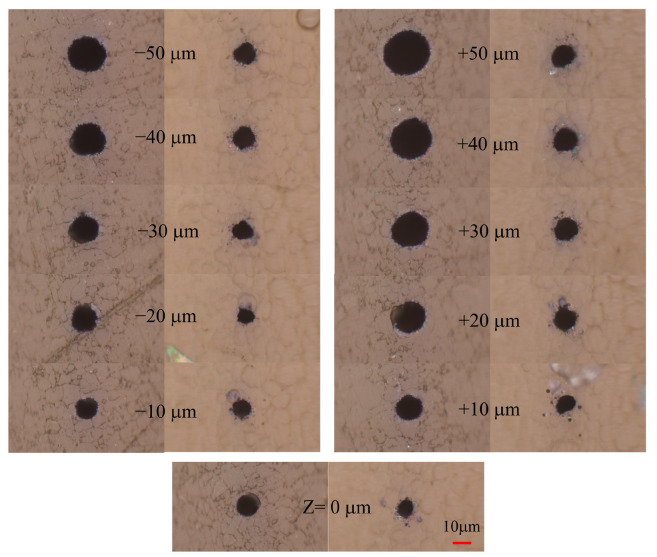
A pulse sequence consisting of three subpulses with $t_{d}$ equals 0.3 ps, generated by a sine phase, was used to drill holes in the GDP film. The morphology of the entrance and exit holes was obtained under different focusing positions after interaction with 60,000 laser shots.

Femtosecond laser microhole drilling in materials is significantly influenced by the focusing conditions. Unlike metals, GDP is a transparent material, allowing the laser to penetrate from its surface and propagate deeper into the bulk. When the laser energy exceeds the ablation threshold, energy deposition can lead to ionization and structural damage inside the material, thus inducing explosive boiling and multiple filamentation of the subsurface during the interaction process [13,16,20,34]. Moreover, at high laser intensities, transparent materials exhibit an intensity-dependent refractive index due to nonlinear optical effects. These nonlinearities modify the dispersion and propagation behavior of the laser pulse within the material [20,39]. In transparent GDP films, the relative position of the laser focus with respect to the sample surface (denoted as Z) plays a critical role in the ablation process. Here, Z < 0 indicates that the laser is focused under the sample surface (inside the material), while Z > 0 means the focus position above the surface. We investigated how this relative focal position affects microhole formation, as illustrated in Figure 3. The position of the sample along the optical axis was precisely controlled using a three-dimensional translation stage, enabling accurate adjustment of the focal depth relative to the sample surface.

**Figure 4:**
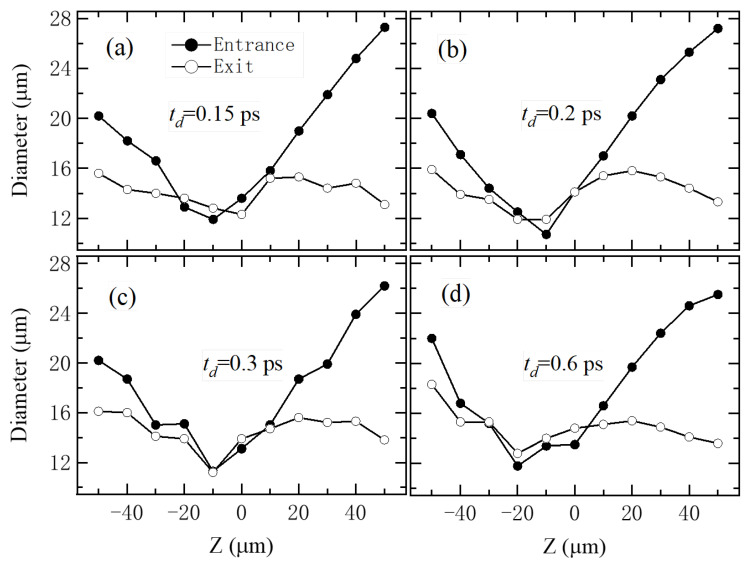
The entrance and exit diameters of microholes drilled with different-shaped pulses (60,000 laser shots) as a function of Z. (**a**–**d**): The results obtained with the TP lasers when $t_{d}$ equals 0.15, 0.2, 0.3, and 0.6 ps, respectively.

To study the effect of Z on microhole fabrication under shaped femtosecond pulses, transmission signals were collected in real time at various Z positions ranging from −50 µm to +50 µm to monitor the dynamic drilling process. As discussed in the previous section, the temporally shaped pulse (TP) with a subpulse interval of 0.3 ps demonstrated an excellent balance of drilling performance, achieving a small hole taper while maintaining high drilling efficiency compared to other pulse shapes. Therefore, only this pulse configuration was used for the Z-scan measurements. Figure 3 presents the microhole morphologies obtained at different Z values, incremented in 10 µm steps. Varying the focus position led to holes with different diameters and shapes. The smallest entrance and exit hole diameters, with minimal differences, were achieved at Z= −10 µm. These observations suggest that ablation initiates within the film, where the laser energy is initially coupled to the electrons, and then the breaking of the C–C and C–H bonds of GDP occurs after electrons transfer their energy to the carbon and hydrocarbon chains through electron–vibration coupling [34]. The resulting plasma introduces spatially and temporally dependent attenuation of the laser intensity [40], which in turn affects the subsequent ablation dynamics. Figure 4 shows the measured entrance and exit diameters of the microholes as a function of Z under four different subpulse intervals: 0.15, 0.2, 0.3, and 0.6 ps. The results show that smaller hole diameters are achieved when the laser is focused inside the film (Z from −10 to −20 µm). In particular, for a subpulse delay of 0.3 ps at Z = −10 µm, the entrance and exit diameters were 11.3 µm and 11.2 µm, respectively, with a minimal difference of just 0.1 µm, as shown in Figure 4c. Compared to the transform-limited pulse, the use of a pulse train with multiple subpulses resulted in smaller and more uniform holes, as indicated by the difference between the entrance and exit diameters.

### 3.3. The Processing Efficiency Under Different Pulse Shapes

**Figure 5:**
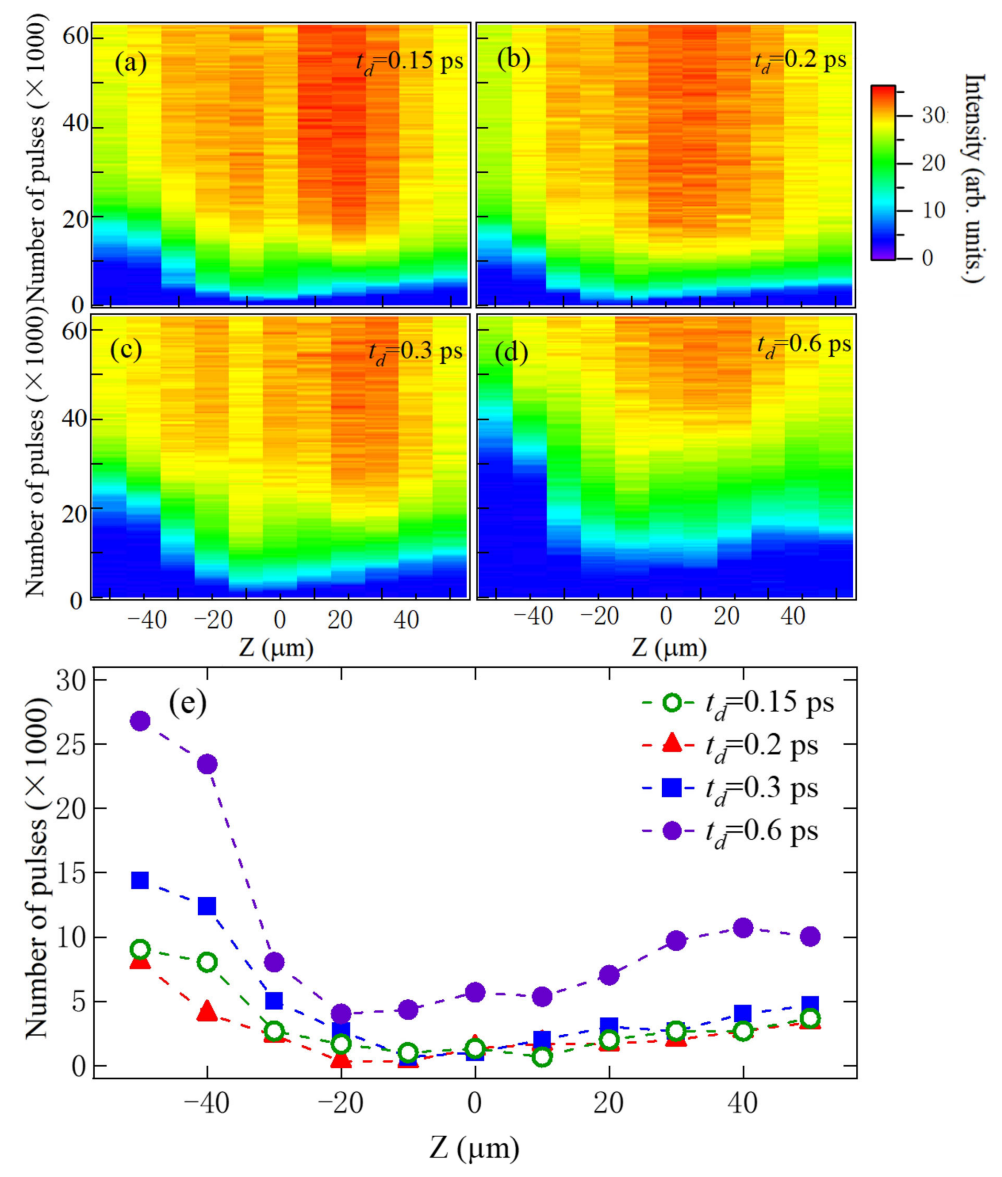
(**a**–**d**): The measured intensity of transmission signals during the drilling process with the photodiode behind the sample when $t_{d}$ equals 0.15, 0.2, 0.3, and 0.6 ps at different focusing positions. (**e**): The minimal number of laser pulses required for drilling through GDP films when $t_{d}$ equals 0.15, 0.2, 0.3, and 0.6 ps at different focusing positions.

To further examine the impact of femtosecond pulse shaping on the processing efficiency of GDP films, we measured the transmission signal using a photodiode as a function of the number of laser pulses. High processing efficiency requires precise optimization of pulse shaping, energy distribution, and focusing conditions to enhance laser–material coupling and promote efficient localized energy deposition. The experimental results are presented in Figure 5a–d, which show measurements taken at different focal (Z) positions for three-pulse sequences with four distinct subpulse intervals. Processing efficiency was quantified by the number of pulses required to completely drill through the GDP film, with fewer pulses indicating higher efficiency. The minimal number of pulses required for drilling through was determined by monitoring the transmission signal. This number was identified as the intersection of two linear fits: one applied to the constant baseline signal before drilling through and another applied to the linearly increasing transmission signal after drilling commenced.

The data clearly demonstrate that both the focal position and pulse shape strongly influence the transmission signal and, consequently, the processing efficiency, as summarized in Figure 5e. When the laser is focused near the sample surface (Z = 0), ablation proceeds rapidly, and the transmission signal increases sharply after only a few pulses (Figure 5a–d). This can be attributed to the strong nonlinear absorption and rapid free-electron generation that occurs when the laser intensity is highest at the focal plane. The resulting energy deposition leads to efficient photoionization and material removal via Coulomb explosion or ultrafast phase transitions. Moreover, the efficiency is highly sensitive to the temporal spacing between subpulses. When the subpulse interval increases from 0.3 ps to 0.6 ps, a significant drop in drilling efficiency is observed (Figure 5e). Physically, shorter subpulse intervals favor constructive energy accumulation within the electron–lattice relaxation timescale (typically a few picoseconds in dielectrics or polymers), allowing the initial subpulse to pre-condition the material (e.g., by generating seed electrons or inducing transient defect states), which enhances absorption of subsequent subpulses. In contrast, longer delays allow partial energy dissipation, reducing the synergistic effect of the pulse train and leading to less efficient ablation. These results indicate that optimizing subpulse timing within the ultrafast energy relaxation window is crucial for maximizing both ablation rate and the quality of material removal.

Efficiency in femtosecond laser processing is a critical parameter as it directly impacts material removal effectiveness, machining precision, and overall process performance [41,42,43]. High ablation efficiency ensures optimal utilization of laser energy, enabling the desired material removal while minimizing heat diffusion and associated thermal damage to surrounding regions. Notably, the highest efficiency was achieved when the laser was focused inside the film (Z < 0), requiring fewer pulses for drilling through than when focused above the surface (Z > 0). This can be attributed to the deposition of more concentrated energy at the focal point within the material, which leads to more rapid ablation starts from inside due to the plasma formation after strong field ionization. The energy transfer and redistribution within the material can be effective, thus breaking the bonds and resulting in faster ablation [13]. Specifically, the optimal drilling efficiency was achieved at Z = −10 µm for subpulses with delays of 0.15, 0.2, 0.3, and 0.6 ps, as shown in Figure 5e. The dependence of efficiency on subpulse delay can be understood in terms of plasma formation and the subsequent energy absorption following the initial subpulse. The reshaping of the next laser pulse after passing through plasma induced by the previous one will reduce the ionization possibility, thereby suppressing the ablation process [40]. Shorter delays allow subsequent pulses to interact more effectively with the partially ionized region, enhancing energy deposition. However, for delays of 0.6 ps or longer, the expanding plasma plume from the initial pulse may obstruct the propagation of subsequent subpulses more strongly, reducing energy coupling and processing efficiency [44].

These findings suggest that maintaining a subpulse interval below 0.3 ps enables high drilling efficiency and promotes the formation of clean symmetric microholes in GDP films. Physically, this improvement can be attributed to enhanced energy coupling between the laser pulses and the material within the characteristic timescales of electron–phonon and electron–lattice relaxation. When subpulses arrive within a few hundred femtoseconds of each other, the material remains in a highly excited non-equilibrium state—characterized by a dense population of free electrons and transient defects—which significantly increases its absorption for subsequent pulses. This cumulative effect promotes efficient ionization and rapid energy deposition, leading to localized ablation with minimal thermal diffusion and mechanical damage. These results demonstrate that femtosecond pulse shaping not only enhances processing efficiency but also enables precise control over the ablation process. By tuning the temporal structure of the pulse train, one can selectively manipulate the ultrafast dynamics of energy absorption, electron excitation, plasma formation, and material response. This capability offers a promising pathway for advancing high-precision micro-machining in soft materials like GDP, where conventional thermal processing often leads to poor edge quality or collateral damage. Further investigation into the fundamental ablation mechanisms—such as multiphoton and tunnel ionization, cascade (avalanche) ionization, plasma expansion dynamics, ultrafast melting, and Coulomb-driven material ejection—is essential for building accurate models of laser–GDP interaction. Such models will guide the optimization of ultrafast laser parameters and pulse sequences, ultimately advancing applications in inertial confinement fusion target fabrication and other areas of precision laser processing.

## 4. Conclusions

To conclude, this study highlights the significant impact of femtosecond laser pulse shaping and the focus position on the ablation and microhole fabrication of GDP films. Using shaped femtosecond pulses with multiple subpulses, smaller and more uniform holes can be achieved. The shaped pulse not only enhances precision but also reduces the diameter variation between the entrance and exit of drilled holes. Additionally, the focusing position of the laser within the material plays a critical role in terms of morphology and processing efficiency. When the laser focus is optimized inside the material, the processing efficiency and quality are improved. This work establishes, for the first time, the critical role of temporal pulse shaping as a novel and effective strategy for precision microhole fabrication in GDP films. By controlling subpulse number and delay, we achieved a superior combination of efficiency and hole quality that is unattainable with standard single-pulse techniques. These findings underscore the potential of tailored femtosecond laser parameters, such as pulse shaping and focus positioning, to achieve highly efficient and precise microhole drilling, with broad applications in microelectronics and material and energy science.

## Data Availability

The original contributions presented in this study are included in the article. Further inquiries can be directed to the corresponding authors.
